# Developing a new academic wordlist for medical purposes – a viable tool for educators

**DOI:** 10.3205/zma001530

**Published:** 2022-02-15

**Authors:** Khalil Tazik, Mahmood Maniati, Mohammad Reza Afshar, Somayeh Biparva Haghighi

**Affiliations:** 1 Ahvaz Jundishapur University of Medical Sciences, Ahvaz, Iran

**Keywords:** medical research article, academic word, technical word, medical students, medical teacher

## Abstract

**Objective:** This study aims at developing a list of academic and technical words commonly used in medical research articles. It is conducted in line with the specificity of academic literacy and vocabulary practices in every individual discipline.

**Methods:** The corpus of this study consisted of 18,462,820 words extracted from 1,784 research articles accessed from three prestigious and widely known journals, i.e., The Lancet, The British Medical Journal (BMJ), and The New England Journal of Medicine (NEJM), published between 2015 and 2019. To analyze the data, the RANGE program as a robust tool for developing viable academic word lists was used.

**Results:** Our quantitative and qualitative data analysis yielded a final academic wordlist which consisted of 1,003 words, covered 1,972,420 words in the corpus, and accounted for 10.68% of the medical research articles.

**Discussion: **The high coverage of the extracted academic and technical words provides a reliable source for medical students, medical educators, material designers, and those who are deeply involved in medical English education.

## Zusammenfassung

**Ziel: **Diese Studie zielt darauf ab, eine Liste von akademischen und technischen Wörtern zu erstellen, die in medizinischen Forschungsartikeln häufig verwendet werden. Es wird im Einklang mit den Besonderheiten der akademischen Alphabetisierung und der Wortschatzpraxis in jeder einzelnen Disziplin durchgeführt.

**Methoden: **Der Korpus dieser Studie bestand aus 18.462.820 Wörtern aus 1.784 Forschungsartikeln, auf die in drei renommierten und weithin bekannten Zeitschriften zugegriffen wurde, nämlich The Lancet, The British Medical Journal (BMJ) und The New England Journal of Medicine (NEJM), veröffentlicht zwischen 2015 und 2019. Zur Analyse der Daten wurde das RANGE-Programm als robustes Werkzeug zur Entwicklung tragfähiger akademischer Wortlisten verwendet.

**Ergebnisse: **Unsere quantitative und qualitative Datenanalyse ergab eine endgültige akademische Wortliste, die aus 1.003 Wörtern bestand, 1.972.420 Wörter im Korpus abdeckte und 10,68% der medizinischen Forschungsartikel ausmachte.

**Diskussion: **Die hohe Abdeckung der extrahierten akademischen und technischen Wörter bietet eine zuverlässige Quelle für Medizinstudenten, Medizinpädagogen, Materialdesigner und diejenigen, die sich intensiv mit der medizinischen Englischausbildung beschäftigen.

## 1. Introduction

Along with studies conducted on the academic vocabularies over different disciplines, various researchers have examined the academic vocabulary items used in medical science research articles. Chen and Ge [4], for example, examined the coverage of Coxhead's [7] AWL (academic word list) on a corpus of medical research articles with 190,425 running words. Their text analysis revealed that AWL accounted for 10.07% of the medical science research papers, and approximately 292 (51.2%) of the AWL word families were present in most of the research articles. They also examined the dispersion of academic vocabulary items across different rhetorical sections of research articles. They reported that academic vocabulary covered approximately 10% of each section of any given research article. Accordingly, they concluded that AWL word families are important for comprehending medical science research articles and have rhetorical functions in medical research articles. However, they noted that the list requires more modification in order to be regarded as a dependable reference for students of medical sciences.

In another study, Wang et al. [32] compiled a corpus of 1,093,011 running words of medical research articles from different online databases. The final list that they provided included 623 word families that accounted for approximately 12.24% of the total tokens in the corpus. This high frequency was claimed to be a firm evidence for the generality and comprehensiveness of their medical academic word list. The authors believed that the list can be used as a guide for designing any academic books for medical students, and instructors can refer to the given words in their phases of language teaching.

In a recent study, Lei and Liu [19] questioned the common procedure used for developing academic word lists (i.e., excluding general words from academic words) and claimed that some general high-frequency words can be included in the academic words as they appear to have special meanings in academic contexts. Therefore, they combined Coxhead's [7] procedure with Gardner and Davies's [12] method to develop a new medical academic vocabulary. They reported that the 819 words that they compiled showed higher coverage of medical texts with 53% shorter word count than the previously established medical wordlist by Wang, Liang, and Ge [32]. 

### 1.1. Why discipline-specific word lists?

According to Hyland and Tse [14], words behave differently across different contexts and disciplines in terms of their frequencies, collocations, and even meanings. In other words, disciplines follow specific literacy because they have different features and divergent epistemologies [8]. These divergent features were previously discussed by North [25] over the reality-oriented writing in hard sciences and rhetorical-oriented features of soft sciences. Therefore, Hyland and Tse [14] concluded that the fruitful way for preparing students is “to provide them with an understanding of the features of the discourses they will encounter in their particular courses” (p.251). Along with this recommendation, various discipline-specific studies have been conducted over different disciplines (e.g., engineering wordlist by Hsu [13] and Mudraya [21]; medical word list by Wang et al. [32]; and nursing academic wordlist by Yang [35], to name only a few). What all these studies have in common in terms of their methodology was the exclusion of general service lists (West [33] or BNC most frequent words), following Coxhead's lead [7]. The major motivation underpinning this practice was that the learners grasp highly frequent general words earlier than academic and technical words [19], [24]. Scholars, such as Gardner and Davies [12], however, questioned this assumption and reported some academic words of Coxhead [7] as general words, and thus blurring the borders of academic and general service words. Moreover, Gardner and Davies [12] maintained that a general word such as “interest” has a different meaning in academic texts compared to its meaning in general texts; likewise, some general words are more frequent in academic texts than in general English. Hence, a basic revision is required for developing new academic wordlists for each discipline. 

Following this method and compiling a corpus of academic and non-academic corpora, Gardner and Davies developed a new academic vocabulary list with a coverage of 13.8% for academic texts, much higher than the coverage reported by Coxhead [7]. 

#### 1.2. Purpose of the study

The present study was devoted to the development of a new academic word list for medical purposes. It was conducted in line with the specificity of academic literacy and vocabulary practices in every individual discipline. 

Additionally, new developments in the area of corpus linguistics and recent studies on the development of new GSL (General Service List) motivated us to develop a new word list for medical students.

## 2. Methods

### 2.1. Corpus compilation

The corpus of this study consisted of 18,462,820 words extracted from 1,784 research articles. The corpus was set large enough to attain desirable findings and, as Krishnamurthy [16] noted, to detect “finer details of language use” (p.175). The articles were accessed from three prestigious and widely known journals, i.e., *The Lancet, The British Medical Journal (BMJ)*, and *The New England Journal of Medicine (NEJM)*, published between 2015 and 2019. The topics covered in these journals included all the specialist areas of the medical research. This advantage helped the researchers to directly focus on the mostly viewed texts for each specialist area. The large number of words included in the corpus and the wide range of research themes included in the journals made our corpus a representative and reliable source for developing a wordlist for medical purposes. 

#### 2.2. Corpus features

1. The corpus composed of all original articles without any format limitation unlike Wang et al. [32][ who selected only original articles which had Introduction, Method, Results, and Discussion sections and excluded others.
2. Review articles were included in the corpus since they are critical in medical education with high readership worldwide.
3. The authors and their mother language were not taken into account since the journals were the top peer-reviewed journals with professional language proofreading processes.
4. The author’s biodata, tables, figures, pictures, graphs, references, appendices, and the points given in non-English languages were excluded from the corpus.

##### Data analysis

To analyse the data, the RANGE program downloadable from [https://www.lextutor.ca/] was used. Nation [24] stated that this software program is a robust tool for developing viable academic word lists. It has been applied in many previous studies [7], [19], [31], [32], [35]. The program consists of three sub-lists: the two-first base lists belong to West's [33] most frequent general words (GSL) and the third base list includes Coxhead's [7] 570 academic word families. After importing any type of corpus, the program analyses the corpus and ranks the words in accordance with their frequency and inclusion in one of these sub-lists. If the word does not belong to any of these base lists, the word is listed as “not in the list”. 

According to the mechanism of the RANGE program and the procedures that researchers follow to develop an academic wordlist, selection of any academic word is based on comparing the lists of words against West’s [33] GSL base lists. However, to update the West’s [33] GSL, Browne et al. [3] and Brezina and Gablasova [2] developed new GSLs derived from a modern corpus. Subsequently, Eldridge [9] believes that the academic lists established according to these newly developed GSLs contain more discipline-specific words expected by Hyland and Tse [14]. The sources of corpus compilation show that New GSL developed by Brezina and Gablasova [2] is more academic-oriented; therefore, in this study, the researchers preferred Brezina and Gablasova [2] as the GSL reference for refining the academic lists. 

The criteria for selecting academic words in this study were:

1. Words that were not included in the GSL [33] and the New General Service List [2] and
2. words that occurred at least 527 times in the entire corpus.

The minimum frequency criterion was duplicated from Coxhead (2000) word selection procedure. Coxhead [7] set the frequency of 100 occurrences in 3.5 million running words, which is about 28.57 times in a million words. This frequency threshold was applied by a large number of studies such as Wang et al. [32], Khani and Tazik [15], and Lei and Liu [19]. 

After quantitative analysis and identifying the words occurring 527 or more times, in the qualitative phase of analysis, semi-words and proper nouns were omitted from the final list. However, frequently used medical abbreviations and hyphenated words were included in the list. The rationale behind this decision was that in medical pedagogy, instructors are seeking the most relevant and useful vocabulary items to be included in their teaching materials, whether they are common single words or abbreviations and hyphenated words. 

Another important issue was comparing the final wordlist against the New General Service List [2] not included in the RANGE program. In this phase of analysis, all words that had a frequency of occurrence of 527 or higher in the entire corpus (whether included in Coxhead’s wordlist or not included in any list) were searched for any possible occurrence in the General Service List. Words that appeared in the given list were excluded from the academic words. In this way, all words would be filtered, yielding the most reliable list to be used as academic medical words. Of course, the exclusion of general words was done with great caution. For example, although the noun form of *statistic* is a general word, it was found that its adjective and adverb forms are academic words, occurring 12546 times in the total corpus. Therefore, they were added to the finally reported academic wordlist. 

To report the remaining academic words, Coxhead [7] organized her report around the word families, assuming that the most frequent form of the word mirrors the importance of the word itself and its derivational forms, and understanding other forms of the word requires little effort. For instance, *inhibit* is the headword for* inhibited, inhibiting, inhibition, inhibitions, inhibitor, inhibitors, inhibitory*, and *inhibits*. Though highlighting the word family has its own advantages, understanding all the derived forms requires extra knowledge of morphology, which is beyond the students' current level [2]. Furthermore, students seek the best way for decreasing reading comprehension problems, and introducing the most frequent word forms is the most viable response to this challenge. Therefore, the most frequent word types rather than word families were the final reported words.

During the analysis, it was found that some headwords in Coxhead’s [7] wordlist did not qualify for the medical academic wordlist; however, careful analysis of their members showed that some family members met the criterion. For example, the word *occurred* only 142 times in its simple form, and based on word family analysis it had to be removed from the final list. However, after analysing its family members, it was observed that the past form of this verb as well as its noun and adjective forms occurred 3275, 1248, 1243 times, respectively. Hence, we concluded that focusing on the headwords or frequency of word family was not a reliable criterion for making decisions on academic word selection. As a result, we lemmatized all the headwords and checked their frequencies one by one. Along with checking the frequencies, the words were also compared against the GSL [2], and word forms regarded as general words were removed from the list. At the end, those words, which met the criterion but were not listed by Brezina and Gablasova [2] were included as academic words for medical research articles. 

After identifying academic words which were shared with Coxhead’s [7] AWL, we started analysing the words that met the frequency criterion but grouped under “not in the list”. In this phase of analysis, the words were included into academic and technical lists. Distinguishing academic words from technical words was done based on group discussions and consulting experts and dependable dictionaries. In cases of any discrepancies, the words were spotted in the context in which they had been used (the article), and their functions were detected. This process continued until final decisions were made. 

## 3. Results and discussion

A total of 232 out of the 570 words families met the criterion to be selected as medical academic words. However, as noted above, some forms of these words were reported to be among the general word list introduced by Brezina and Gablasova [2]. Moreover, one of the pitfalls of selecting words based on word family is that the frequency of the headwords is the criterion but occurrences of their subheads (other parts of speech) are not considered by the RANGE program. Therefore, in order not to miss these word types and to remove the general words from the academic list, all the qualified word families and word types, which occurred more than 527 times, were lemmatized and checked against the New GSL. After lemmatizing and checking this list against the newly developed GSL [2], 941 word types were removed from the list and 174 word types were selected as academic words for medical RAs (research articles). These words occurred 404,066 times, accounting for 2.18% of the total corpus. This process showed the importance of renewing the academic words for medical purposes and updating studies of such purposes. In fact, it showed that Coxhead's [7] academic word list is unequally valuable for all fields of study, and medical students’ needs are different. This need for differentiation is evident from the coverage of Coxhead’s [7] wordlist in the entire corpus. Also, it can be argued that updating West’s [33] GSL and teaching New GSL by Brezina and Gablasova [2] can be a fruitful prerequisite for comprehending academic texts. Without taking these points into account, the students may consider general words as academic or technical words and look up a huge number of general words in medical dictionaries, which is a time-consuming process. 

After identifying the academic words, which were shared with Coxhead [7], it was found that although some words had the frequency requirements for being considered as academic words, they were not among Coxhead’s [7] academic words. We put these words in a separate table, and ranked them according to their frequency. Then, we determined whether these words are academic, technical, or general. To start with, a group discussion involving the four authors of this study along with three MDs (doctors of medicine), who had taught English for Medical Purposes (EMP) for some years, was held. During this session, the words were spotted in the context in which they appeared and the participants of the group discussion looked the words up in the dictionaries and commented on the class that the words belonged to. In fact, the functions and uses of the words were the basis of the group decisions. In case of any discrepancies, the final decisions were made by the more experienced members. After the discussion session, out of the total 868 words not found in any list, 39 words (e.g., *patient, cancer, clinical, score, drug, infection, surgery, permission*, etc.) were considered general and removed from the list, 820 word types were considered technical words, and the rest (9 words) were considered academic words. Therefore, the refined list included 829 words, which occurred 1568354 times and accounted for 8.49% of the total corpus. These 829 new words were added to the list of word types shared with Coxhead [7] (see table 1 (Tab. 1)). 

The final academic wordlist consisted of 1003 words (see attachment 1 ), covering 1,972,420 words in the corpus (see table 1 (Tab. 1)). Each word in this list occurred about 106 times per 1000 words. This impressive figure shows the significance of the obtained list, especially compared to the wordlist introduced by Wang et al. [32]. The new medical academic wordlist developed in this study covers 10.68% of the medical science corpus. The efficacy of this coverage, when compared to related studies, becomes clearer. As noted by Laufer [17], given this high coverage, if medical students master these lists of words, they will be able to comprehend the medical texts (at least articles in medical journals). 

### Coverage across the corpus

The coverage of the wordlist developed in this study was compared to the wordlists established by Wang et al. [32] and Lei and Liu [19] (see table 2 (Tab. 2)).

As can be seen from the table, the coverage of academic and technical words in our study is slightly more than that of Wang et al. [32] and considerably less than that of Lei and Liu [19]. To demonstrate the difference between the three medical wordlists, take this excerpt which is a part of an article from BMJ journal published in 2018. It provides a context for the occurrences and coverage of new medical academic word list in the medical texts. The compatibility of the previously developed wordlists and the differences they have with the given wordlist in this study is clearly provided in this example. 

Among the findings for cancers* at specific* sites* (see table 2 (Tab. 2)), 25-hydroxyvitamin D concentration* showed a significant* inverse association* with the risk* of liver cancer* (P for trend=0.006). Further adjustment for dietary factors* such as **intake** of total energy, fruits and vegetables, meat, fish and shellfish, isoflavone, green tea, and coffee slightly attenuated the association*, but it remained significant*, with **hazard** ratios from the second to fourth quarters compared with the first quarter of 0.79 (0.48 to 1.28), 0.71 (0.43 to 1.18), and 0.51 (0.29 to 0.90), **respectively** (P for trend=0.02). In a **subset** of the population* with available information, further adjustment for hepatitis B and hepatitis C virus infection* **status** and alanine aminotransferase concentration* in the multivariable model did not appreciably **alter** the association* of 25-hydroxyvitamin D with liver cancer*.

In this 130-words excerpt, academic words found in this study were underlined, Lei and Liu’s [19] academic words were bold typed, and Wang et al.’s [32] were marked with an asterisk. As it is shown, 13 (10%) words were underlined, 22 (16.92%) words bold typed, and 16 (12.30%) words were asterisked. It can also be seen that only 4 academic words of this excerpt are repeated in both our list and that of Lei and Liu’s [19] study. This difference is related to the word selection procedure used by Lei and Liu [19] and their tendency to ignore technical words. Interestingly, all words identified in Wang et al.’s [32] wordlist were shared with Lei and Liu’s [19] wordlist, and all these words were reported by Brezina and Gablasova [2] as general words. If students only rely on the two previously developed wordlists, they will miss precious and influential words, which are highly effective in understanding medical RAs. Therefore, medical students refer to the new GSL [2] before writing or studying RAs. In this way, they could bring this basic knowledge to class and by studying the most frequent academic and technical words presented in this study, they will be equipped with the academic and technical knowledge that is essential for understanding the whole RAs. Of course, we do not underestimate the scientific values of other wordlists, and they can be still used alongside the list developed in this study. In cases of any problems students have with the general or academic uses of words, they can refer to other wordlists and spot the challenging words. 

According to the above-mentioned discussions, it could be argued that this wordlist could safely serve the medical students better than previously developed lists because of its time-effectiveness for learning, confidential representation of different ranges of topics and subfields of the medical science, and its higher coverage in terms of both academic and technical words. Another advantage of this wordlist is the exclusion of general words and inclusion of technical words in the analysis. The comparison of the list with other lists shows that the coverage of this list is satisfactorily higher.

Research articles are the most accessible and up-to-date sources of knowledge. All medical educators motivate their students to read these sources in order to keep their knowledge in line with the medical findings and trends of research in interdisciplinary areas. The list of academic words developed in this study will make the way for easier understanding of the texts, especially for non-native students, and it highlights the most appropriate words for teaching in general and specific courses. Accordingly, medical educators can find this list as a guide for their students and, instead of teaching less frequent words, stick to the most frequent and important academic and technical words that are essential for comprehending research articles or any other medical texts. 

## 4. Educational implications and conclusions

The vocabulary items listed in the new medical wordlist (NMWL) evidence the significant role of this list for the medical students and the members of this discourse community. In line with the discipline-specific studies, the NMWL approves that the words occur and behave differently across different fields of study, generating new concepts for disciplinary academic literacy. This specificity of the word list helps academic material developers to develop materials that are both scientifically scrutinized and experimentally attested, which can be of paramount help to students of medicine. This strand of material development is noted by Murray [22] who acknowledges that by understanding the academic demands of students studying in discrete disciplines, instructors “can research appropriate materials and produce relevant lessons and engaging, and which, therefore, promote learning most effectively” (p.3). Therefore, medical teachers can use the NMWL to provide the most relevant and appropriate teaching materials. Moreover, by using NMWL, medical students will be equipped with a wordlist directly derived from the written scientific medical discourse, enabling them to study research articles independently. 

The high coverage of the academic words extracted in the present study should lead to the development of reliable sources for medical students, teachers, material designers, and those who are deeply involved in medical English education. As noted earlier, the list includes academic words, technical words, most frequent acronyms and abbreviations, and the general words that have special meanings in medical texts. It is recommended that students interested in learning the items of NMWL, first refer to the general words, and then try grasping academic and technical words. In this way, they will be equipped with prerequisite knowledge for understanding medical research articles. 

According to the results of the present study, medical English teachers should bring in a medical dictionary to the class for teaching medical academic and technical words if the functions and uses of words in medical contexts are the final aims for comprehending medical texts. Of course, general meanings of the words are also needed to be reviewed, but the major focus should be on the medical uses not the general ones. Since the NMWL includes the high-frequent technical terms, the students will be more encouraged to take the wordlist into serious account and have frequent consultations with it. A question might be posed here about the use of such a wordlist when comprehensive medical dictionaries are already available. The answer lies in the frequency of technical terms in research articles, and that not all technical terms have equal learning value. Students need to learn the most frequent and usable words, rather than cramming their mind with unnecessary items that occur in medical texts once in a blue moon. Moreover, aside from its uselessness, grasping a dictionary of thousands of technical terms is an impossible undertaking, which takes a noticeable amount of time and energy. 

Another important issue for teachers to take into account is related to locations of the listed words. Teachers should provide opportunities for medical students to spot word collocations, since researchers such as Hyland and Tse [14] hold that words collocate in distinct ways and having information about these distinct ways is valuable and even mandatory for learning the uses of academic words in special contexts. 

## Funding

This project [U-98249] was conducted under the support of Ahvaz Jundishapur University of Medical Sciences, Ahvaz, Iran, and approved by the Ethics Committee of this university (Ref. ID: IR.AJUMS.REC:1398.84).

## Competing interests

The authors declare that they have no competing interests. 

## Supplementary Material

Academic and technical words in alphabetical order

## Figures and Tables

**Table 1:**
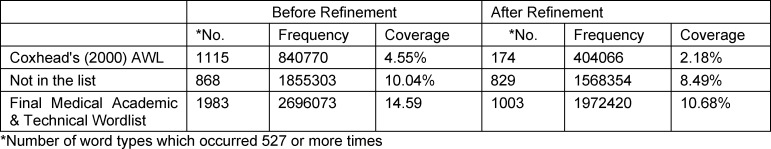
Refinement of academic word lists

**Table 2:**
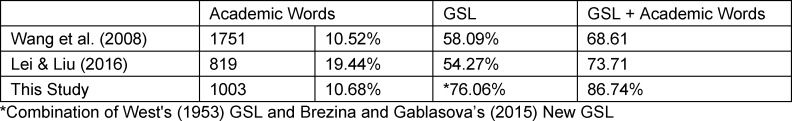
Coverage of academic words and GSL across three corpora
