# Supplementary material for: Developing a new academic wordlist for medical purposes – a viable tool for educators
Source: GMS J Med Educ. 2022 Feb 15;39(1):Doc9. doi: 10.3205/zma001530 (PMC8953199; doi:10.3205/zma001530)
Supplement: Academic and technical words in alphabetical order [file JME-39-9-s-001.pdf]

**Attachment 1:** Academic and technical words in alphabetical order

Bold words are in common with Lei and Liu (2016)/ * words are in common with Wang et al. (2008)

**A: Abdominal***; Ablation; **Abnormalities**; Abortion; Abortion; Abstinence; Abstract(s) *; Abuse; Ace; **Acid*;** Acknowledgment; **Acute;** Adapt***;** Additionally; ADHD (Attention-deficit/hyperactivity disorder); Adherence; Adiposity; Adjuvant; Administered; Administrative; Admission(s); Adolescent(s); Adverse; Affirms; Ageing; Aggregate; **Agonists**; **Albumin**; Algorithm; All-Cause; Allergy; Allocated; Allocation; **Alter***; Altman; Alzheimer; Ambulatory; Anaemia; Anaesthesia*; Aneurysm; Angina; Angiography; Angiotensin; Ankle; Anomalies; Antagonist; Antenatal; **Anterior**; **Antibiotic*; Antibody(ies)**; Anticoagulant; Anticoagulants; Anticoagulation; Antidepressant(s); Antigen; Antihypertensive; **Anti-Inflammatory;** Antiplatelet; Antipsychotic; Antiretroviral; Aortic; APGAR (Appearance, Pulse, Grimace, Activity, and Respiration); Appendix(ces); **Arterial; Artery;** Arthritis; Arthroplasty; Ascertainment; Aspirin; **Assay*;** Assigned***;** Assignment; Assumption(s); Asthma; Astrazeneca; **Asymptomatic;** Atherosclerosis; Atrial; Attenuated***;** Attributable; Attributed***;** Attribution; Audit***;** Autism; Automated

**B: Bacterial**; Balloon; Bariatric; **Baseline*; Benign**; Benzodiazepine(s); Beverages; Bias(ed) ***;** Binary; Biobank; **Biological**; Biomarkers; Biomedical; **Biopsy*;** Biostatistics; Bipolar; Birthweight; Bladder; Blocker(s); BMI (Body Mass Index); Body-Mass; Borderline; **Bowel**; BP (Blood pressure); **Burden;** Bypassc.

**C:** Caesarean; Calcium***;** Calendar; Calibration; Capita; Capture*; **Carcinoma**; **Cardiac**; Cario; Cardiology; **Cardiovascular**; Carers; Carotid; Case-Control; Categorical; Catheter***;** Causal; **Cell**(s) ***;** Censored; Census; Centile; **Cerebral;** Cerebrovascular; Cervical; Cessation; Chemotherapy; Chlamydia; Cholesterol; Chronic*; CI (confidence interval cardiac index); **Circulating; Circulation*;** Circumference; Cirrhosis; Clinic(s) *; **Clinically**; Clinicaltrials; **Clinician**(s); Clopidogrel; **Cluster**(s)***;** Clustering; Cochrane; Coefficient(s); **Cognitive***; **Cohort*;** Colitis; Collaboration; Collaborative; Colon; Colonoscopy; Colorectal; Commission; Communication; Comorbidity(ies); Comparator; Complemen(s); Complementary; Complementation; **Compliance**; **Compute**(d); Concealment; Conceived; Conception; Concomitant***;** Concurrent; Confounders; Confounding; Congenital; Congestive; Consecutive; Consent*; Conservative; Consort; Consortium; Consulting; Contraception; Contraceptive(s); Convert(ing); COPD (Chronic obstructive pulmonary disease); Cord*; Coronary; **Correlated**; **Correlation**; Correspond(ing) *; Corticosteroid(s); Cost-Effectiveness; Counselling; Covariate(s); CPRD (Clinical Practice Research Datalink); Creatinine; creative; Criteria(ion*); Crossover; Crude; CT (Computed tomography); Cumulative; Cut-off ; CVD (cardiovascular disease); Cytology;

**D:** Dabigatran; Dairy; DAPT (Dual antiplatelet therapy); Database(s); Dataset(s); D-Dimer; Deemed Defects*; Deficiency; Definite; Delirium; Dementia; Demographic(s); Density; Depressive; Depressive; Deprivation; Deprived; Derivation; Derivative; Determinants; Developmental; **Deviation**(s); DF (degrees of freedom); **Diabetes**; Diabetic; **Diagnosed*; Diagnosis; Diagnostic**; Dialysis; **Diameter*** Diarrhoea; Diastolic; **Diet*;** Dietary; **Differential**; **Discharge*;** Discharged; Disclosure; Discontinuation; Discontinued; Discrepancies; Discrimination; Distress; Diuretics; dm (diabetes mellitus); DNA (Deoxyribonucleic acid); **Documented*;** Donor*; **Dose*;** Dose-Response; Dosin; Double-Blind; Draft(ed); Drug-Eluting; Dual; Durable; **Dysfunction**;

**E:** Ebola; Efficacy*; Elective; Electronic; **Elevated**; **Elevation***; Eligibility; Eluting; EM (Emergency medicine); Embase; Embolism; Embryo*; Emergency; Emergent; Empirical; Endocrinol; Endometrial Endovascular; Endpoint(s); Enrolled*; Enrolment; Environ; Enzyme; Epidemic; **Epidemiological**; **Epidemiology**; Epidural; Epilepsy; **Equivalent**; ER (Emergency room); Ethic ; Ethical; Ethnicity; Exacerbation(s); Excision; Expenditure; **Exposure**(s); Extracted*

**F:** Faculty; Faecal; Fasting; Fatal; Fatality; Fatigue; FDA (Food and Drug Administration); Feasibility; Feasible; Feedback; Femoral; Fetal; FEV (Forced expiratory volume in one second) Fibre; Fibrillation; Fibrosis; Fifths; Filtration; **Fluid***; Folic; **Follow-Up*;** Formula*; **Fraction***; Fracture(s); Funder(s); Funnel;

**G:** Gastric; Gastroenterology; Gastrointestinal; Gender***; Gene***: Generalisability; Generic; **Genetic**(s); **Genotype**; Geographical; Geriatric; Gestation; Gestational: **Global;** Glomerular; **Glucose*;** Glycaemic; GP (General physician); **Grade***; Graduates; Graft***;** Guarantor; **Guideline**(s) ***;** Gynecol;

**H:** HN (Hemagglutinin Type and Neuraminidase Type) Haemoglobin; Haemorrhage; Haemorrhagic; **Hazard**(s); HCV (hepatitis C virus); Headache; Healthcare; Health-related; Height; Hemorrhage; Heparin; Hepatitis; Heterogeneity; Hg; High-Income; High-Risk; Hip; HIV (human immunodeficiency virus); Hormone; HPV

Attachment 1 to Tazik K, Maniati M, Afshar MR, Haghighi SB. *Developing a new academic wordlist for medical purposes – a viable tool for educators.* GMS J Med Educ. 2022;39(1):Doc9. DOI: 10.3205/zma001530

(human papilloma virus); HR (heart rate) Hygiene; **Hypertension***; Hypertensive; Hypoglycaemia; **Hypothesis*;** Hysterectomy

**I:** ICD- (International Classification of Diseases, Tenth Revision); ICU (intensive care unit); **Imaging**; **Immune**; Immunisation; Impaired; Impairment; Implant; Implantation; Implementation; Implication(s); Imputation; Imputed; **(In)Adequate*; Incidence**(s)***;** Incremental; Index; **Induced*;** Inequalities; Infant(s); Infarction; Infect***;** Infected; **Infectious**; **Inflammation***; **Inflammatory;** Influenza; **Infusion**; Inhaled; **Inhibition**; **Inhibitor**(s); In-Hospital; **Initially**; **Initiated**; **Initiation**; Initiative; **Injection**(s); Inpatient; Institute(s); **Insulin**; **Intake**(s) *; Integrated***;** Integrity*; Intellectual Intensity; Intensive; Intention-To-Treat; Interim; **Intermediate**; Intermittent; Intern; **Interpreted*** Interquartile; **Interval**(s); **Intervention**(s); Intracerebral; Intracranial; Intraepithelial; Intrauterine; **Intravenous**; **Invasive;** Inverse; Ischaemia; Ischaemic; **Isolated***; IV (intravenous); IVF (in vitro fertilization);

**J:** Journal(s);

**K: Kidney**;

**L: Laboratory** *; Lag; Laparoscopic; Laser*; LDL (low-density lipoprotein)**; Lesion**(s); **Linear* Linkage**; Lipid***;** Lipoprotein; Lisense(s); Liver***;** Logistic*; Longitudinal; Low-Income; Lumbar; Lymphoma;

**M: Magnetic*;** Magnitude***;** Malaria; Malformations; **Malignancy*;** Mammography; Manual***;** Manuscript; **Margin*;** Masked; Masking; Maternal; Measles; Media; Median; Medicaid; Medicare; Medication(s); Melanoma; **Mellitus**; Merck; Mesh; **Meta-Analysis**; Metabolic***; Metabolism**; Meta-Regression; Metastatic; Metformin; Methadone; Methotrexate; Mg (milligram); MH (Malignant hyperthermia); MI (myocardial infarction); Migraine; **Migration**; **Minimal**; **Minimize*;** Minimum; Ministry; Miscarriage; Misclassification; Misuse; Mmol (one thousandth of a mole ) Mobile (n); **Modified*;** Mol (Mole); **Molecular**; Monotherapy; **Morbidity***; **Mortality*;** MRI (Magnetic resonance imaging); Multicentre; Multivariable; Multivariate; Musculoskeletal; Mutation*(s); Myocardial;

**N:** NA (Natrium); Nasal; Nausea; **Neonatal**; Neoplasia; **Nerve; Neurological**; Neurology; Newborn; NHS (National Health Service); Nicotine; Non-Commercial; Non-Commercially; Non-Communicable; Non-Fatal; Non-Inferiority; Non-Significant; Non-Steroidal; Novartis; Novel; NP (Nasal Passage); NSAIDS (Non-Steroidal Anti-Inflammatory Drugs); Null; Nutrition; Nutritional;

**O:** Obese; **Obesity**; Objective*(s); Observational; Obstet; Obstetric(s); Obstructive; Occupation; Odd***(**s); Oestrogen; Offspring; Oncology; Ongoing; Onset*; Open-Label; Opioid(s); **Optimal*; Oral*;** Orthopaedic; Osteoarthritis; Osteoporosis; Outpatient; Ovarian; Overdiagnosis; Overdose; Overview; Overweight; **Oxygen*;**

**P:** PA (physician assistant); Paediatric; Palliative; Palsy; Pancreatic; Pancreatitis; Pandemic; Papillomavirus; Paracetamol; Parallel***;** **Parameter***(s); Parity; Parkinson; Particulate; **Pathology***; **Pathway***(s), PCI (Percutaneous coronary intervention, also coronary angioplasty); **PEAK*;** Podiatry; Pediatric(s); Peer; Pelvic; Percutaneous; Perinatal; Perioperative; Peripheral; Per-Protocol; **Persistent**; PH (power of hydrogen); Pharmacy; Pharmaceutical(s); Pharmacoepidemiol; Pharmacological; Pharmacology***;** Pharmacy; **Physician**(s); **Physiological**; Physiotherapy; Pilot; Pioglitazone; **Placebo**; Placebo-Controlled; **Plasma*;** Platelet; Plot*; PM (particulate matter); PMID (PubMed reference number); Pneumococcal; Pneumonia; Pollution; Polymer; Pooled; Population-Based; Posterior; Postmenopausal; Postnatal; Postoperative; Postpartum; Potassium; PPI (proton pump inhibitor); Practitioner(s); Pragmatic; Predefine; Predefined; **Predictive**; Predictor(s); Pre-eclampsia; Pre-Existing; Pregnancy(ies); Pregnant; Preliminary*; Premature; Prenatal; Preoperative; **Prescribed***; **Prescription**(s); Prespecified; Preterm; **Prevalence***; **Prevalent**; Preventive; Principal; Priori; **Profile***(s); **Prognosis**; **Prognostic**; **Progression;** Progression-Free; **Prolonged***; Propensity; Prophylactic; Prophylaxis; **Prospective;** Prospectively; Prostate; Protein***;** Protocol*(s); Proton; Psoriasis; Psychiatric; Psychiatry; **Psychological**; Psychology; Psychosis; Psychosocial; Pulmonary; Pylori;

**Q: Qualitative*;** Quantitative**;** Questionnaire*(s); Quit;

**R:** Radiation; Radiographic; Radiotherapy; **Randomised**; Randomization; Randomized; **Ratio***(s); RCT (randomized controlled trial); **Reactive**; Readmission(s); Recall*; **Receptor*; Recipients**; **Recover;** Recruited*; Recruitment; Rectal; Recurrence; Recurrent; **Referral**; **Regimen**(s); Registries; Registry; Regression*; **Regulation**; **Regulatory**; Rehabilitation; Relapse***;** Remission; Remix; Remote; **Removal**; **Renal*;**

Reproductive; Resection; Resident(s); **Residual*; Resistant**; Respiration; **Respiratory*;** Resuscitation; Retinopathy; Retrieved; Retrospective; Reuptake; Reuse; Revascularisation; Reverse*; Reviewers; Revised; Revision; Rheumatoid; Rheumatology; Rituximab; Rivaroxaban; RNA (Ribonucleic Acid); Robust*; Rotavirus; Routine; Routinely;

**S:** Saturated; Scan*(s); Schedule*(d); Schizophrenia; Sclerosis; SD (standard deviation); Sectional; Seizure; **Selective**; Sepsis; **Sequence*;** Sequential; Serotonin; **Serum**; Sessions; Sham; Sided; Smoker; Sociodemographic; Socioeconomic; Sodium; Specialty; Specifically; **Specificity**; Specified; Spectrum***;** Spinal***;** Spine; Sponsor; Spontaneous***;** Squamous; SSRIS (Selective serotonin reuptake inhibitors); **Stable***; Standardised; Statin; Statistical(ly); Stenosis; Stent; **Steroid(s)**; Stillbirth; **Stimulation;** Stratification; Stratified; Subarachnoid; Subcutaneous; **Subgroup**(s); Subjective; Sub-Saharan; Subset; Substitution; Subtotal; Subtypes; Suicidal; Suicide; **Summarised**; **Summary***; **Superior*;** Superiority; Supervised; Supplemental; **Supplementation**; Surg (Surgery); **Surgeon**(s); **Surgical**; Surrogate; **Surveillance**; **Survival**; Survivors; SW (Swab); **Symptomatic**; **Syndrome***(s); **Synthesis*; Systemic**; Systolic;

**T:** Tamoxifen; Technical; **Temporal**; Termination; **Therapeutic**; **Therapy***(ies); **Threshold***(s); Thromboembolism; Thrombosis; Thyroid; **Timing**; **Tissue*;** Tocilizumab; Tolerability; **Tolerance**; **Tomography**; **Toxicity; Tract*;** Tranexamic; Transfer***;** Transfusion; Transient***;** Transparency; Transparent; Transplant***;** Transplantation; **Trauma***; Traumatic; Triage; Trimester; Triple; Tropical; Troponin; Tuberculosis; Tumour*(s);

**U: Ultrasound**; Unadjusted; **Unclear; Undergo***(ing); **Underlying**; **Underwent**; Unexposed; Uniform*; Unmeasured; Unrelated; Unsafe; Untreated; Update; **Uptake**; Urgent; **Urinary**; **Urine**; Uterine;

**V:** Vaccinated; Vaccination; Vaccine(s); Vaginal; Valid***;** Validity; Valve; Varenicline; **Variability; Variable**; **Variant;** Variate; **Vascular***; **Vein*; Venous**; Ventilation; **Ventricular**; Versus***;** Viral; Virus***; Vitamin**; Vitro; Vomiting;

**W:** Ward; Warfarin; Weighted; Wellbeing; Widespread***;** Withdrawal; Withdrew; Worldwide;

**Z:** Zoster
